# 16S rRNA gene-based profiling of the human infant gut microbiota is strongly influenced by sample processing and PCR primer choice

**DOI:** 10.1186/s40168-015-0087-4

**Published:** 2015-06-22

**Authors:** Alan W. Walker, Jennifer C. Martin, Paul Scott, Julian Parkhill, Harry J. Flint, Karen P. Scott

**Affiliations:** Microbiology Group, Rowett Institute of Nutrition and Health, University of Aberdeen, Aberdeen, AB21 9SB UK; Pathogen Genomics Group, Wellcome Trust Sanger Institute, Wellcome Trust Genome Campus, Hinxton, Cambridgeshire CB10 1SA UK

**Keywords:** 16S rRNA gene sequencing, Bifidobacteria, Infant, Intestinal microbiota, FISH

## Abstract

**Background:**

Characterisation of the bacterial composition of the gut microbiota is increasingly carried out with a view to establish the role of different bacterial species in causation or prevention of disease. It is thus essential that the methods used to determine the microbial composition are robust. Here, several widely used molecular techniques were compared to establish the optimal methods to assess the bacterial composition in faecal samples from babies, before weaning.

**Results:**

The bacterial community profile detected in the faeces of infants is highly dependent on the methodology used. Bifidobacteria were the most abundant bacteria detected at 6 weeks in faeces from two initially breast-fed babies using fluorescent in situ hybridisation (FISH), in agreement with data from previous culture-based studies. Using the 16S rRNA gene sequencing approach, however, we found that the detection of bifidobacteria in particular crucially depended on the optimisation of the DNA extraction method, and the choice of primers used to amplify the V1–V3 regions of 16S rRNA genes prior to subsequent sequence analysis. Bifidobacteria were only well represented among amplified 16S rRNA gene sequences when mechanical disruption (bead-beating) procedures for DNA extraction were employed together with optimised “universal” PCR primers. These primers incorporate degenerate bases at positions where mismatches to bifidobacteria and other bacterial taxa occur. The use of a DNA extraction kit with no bead-beating step resulted in a complete absence of bifidobacteria in the sequence data, even when using the optimised primers.

**Conclusions:**

This work emphasises the importance of sample processing methodology to downstream sequencing results and illustrates the value of employing multiple approaches for determining microbiota composition.

**Electronic supplementary material:**

The online version of this article (doi:10.1186/s40168-015-0087-4) contains supplementary material, which is available to authorized users.

## Background

The gut microbiota plays a key role in the maturation of the host immune system, and it is believed that the natural progression in bacterial colonisation of the infant gut is important for health throughout life [1–4]. Early studies of pre-weaned infants showed that the gut microbiota, particularly in breast-fed infants, was dominated by the *Bifidobacterium* genus, which formed as much as 60–90 % of the total microbiota. These findings were based on data from culture methods [5–8] and were subsequently confirmed by studies using molecular techniques such as denaturing gradient gel electrophoresis (DGGE; [9]), quantitative real-time PCR (Q-PCR; [10], and fluorescent in situ hybridisation (FISH; [11, 12]). The abundance of bifidobacteria in stool samples obtained from breast-fed babies correlates with the rich oligosaccharide content of breast milk [13], which is considered to stimulate the growth of *Bifidobacterium* species that possess the capability to utilise these oligosaccharides [14]. Post-weaning, the microbiota gradually changes, with the proportion of bifidobacteria declining as groups of bacteria from the *Firmicutes* and *Bacteroidetes* phyla that are able to utilise complex plant-derived polysaccharides become established [15]; it is currently thought that it may take up to 3 years to establish an adult-like microbiota [16].

Modern techniques, mostly involving targeted sequencing of bacterial 16S rRNA genes or direct metagenomic sequencing, have been used in many studies attempting to define the composition of the healthy adult gut microbiota [17–21]. These techniques have also been applied to the infant gut microbiota. The results from these studies have provided valuable comparisons on the microbial composition in sets of babies with different birth and early nutritional circumstances [22]. However, the lack of standardisation in the methodology used has in some cases resulted in conflicting results, with particular discrepancies in the apparent abundance of bifidobacteria (e.g. [23] versus [24]). Specific comparisons of DNA extraction methods have illustrated the importance of a mechanical lysis step (typically involving bead-beating; [25–27]), while other studies have shown that the choice of PCR primers is critical. For example, the widely used “universal” primers targeting the full-length 16S rRNA gene (27f and 1492r primers) fail to amplify more than 40 % of purified *Actinobacteria* isolates [28]. Previously reported improvements have been to optimise the 16S rRNA gene primer sequences to access the *Bifidobacterium* genus or alternatively to target different genes in order to specifically enumerate bifidobacteria [29–31]. Frank et al. developed variants of the 27f primer *in silico*, which could be used in combination to facilitate amplification of all bacteria in mixed environmental samples [32]. These primers were tested by quantifying bacterial genera in vaginal DNA samples [32]. In contrast, Sim et al. designed degenerate “bifidobacteria-optimised” primers targeting the V3–V5 regions of the 16S rRNA gene [33] and confirmed enumeration of the *Bifidobacterium* genus in infants by comparing pyrosequencing 16S rRNA gene survey data with that obtained by FISH.

In this study, we assessed the microbial profile generated using 454 pyrosequencing of the V1–V3 variable regions of 16S rRNA genes in faecal samples from two babies, comparing the effect of different DNA extraction methods and different amplification primers on the abundance of specific bacterial taxa. We found that the proportional abundance of the *Bifidobacterium* genus only concurred with data estimated by FISH when the FastDNA SPIN Kit for Soil, which includes a mechanical lysis step, was used for DNA extraction, followed by the use of a broadened “universal” forward PCR primer set. In contrast, we show that the widely used 27f primer, which was included in the standard operating procedures released by the Human Microbiome Project [21], and non-mechanical lysis-based DNA extraction kits are sub-optimal for samples containing high levels of *Actinobacteria* and thus cannot be recommended for use with faecal samples, particularly those from infants.

## Results and discussion

### Determination of microbiota composition from 16S rRNA gene sequences is highly dependent on methodology

Previous work has demonstrated that microbial compositional profiles determined using 16S rRNA gene sequencing are subject to several technical/methodological biases [34]. Therefore, we set out to compare the 454 sequence data arising from two different, widely used, DNA extraction methods, and using different PCR primer sets aimed at the commonly targeted V1–V3 variable regions of the 16S rRNA gene.

In method 1, using our standard methodology, DNA was extracted from samples using the FastDNA SPIN Kit for Soil, but we also compared the effect of extending the initial bead-beating time (for mechanical disruption of cells) from the recommended 30 s to 2 min and 5 min time periods. The extraction efficiencies for different bacterial genera were rapidly assessed by quantifying the DNA by Q-PCR, using the generic UniF/R primer sets for all bacteria or specific primers for bifidobacteria, *Bacteroides* and *Lachnospiraceae* (Table 1). The detection of all bacterial groups increased when the cell disruption time was increased from 30 s to 2 min (Additional file 1: Figure S1). A further increase to a 5-min disruption time had little additional effect and was in fact counterbalanced by an associated decrease in detection of other taxa (determined following 16S rRNA gene sequencing; Additional file 1: Figure S2), possibly due to degradation of DNA released from lysed cells during extended bead-beating. For baby N-BF (natural birth, solely breast-fed), 30 s of bead-beating gave lower proportional abundances of bifidobacteria and higher proportional abundances of *Clostridia* and *Firmicutes* than 2 or 5 min bead-beating (Additional file 1: Figure S2b), while the differences for baby C-MF (born by C-section and breast-fed for 4 weeks, fed a mixed bottle/breast milk diet for weeks 5–10; and formula-fed from week 11) were much less marked. Subsequent DNA extractions from infant samples therefore involved bead-beating for 2 min, in 4 × 30 s bursts, with storage on ice between homogenisations.

**Table 1:** Primers used for PCR amplification (and prior to 454 pyrosequencing)

Primer name	Primer sequence	Target group/specificity	Reference
Forward primers			
27f^a^	AG**A**GTT**T**GAT**C**CTGGCTCAG	Universal	Methé et al. [21, 53]
27f-YM^b^	AG**A**GTT**T**GATYMTGGCTCAG	Universal	Satokari et al. [35]
27f-Chl^c^	AGAATTTGATCTTGGTTCAG	Universal/*Chlamydiales*	Frank et al. [32]
27f-Bor^c^	AGAGTTTGATCCTGGCTTAG	Universal/*Borrelia*	Frank et al. [32]
27f-Bif^c^	AGGGTTCGATTCTGGCTCAG	Universal/*Bifidobacteriales*	Frank et al. [32]
27f-Ato^c^	AGAGTTCGATCCTGGCTCAG	Universal/*Atopobium* group	Frank et al. [32]
Bif164-f	GGGTGGTAATGCCGGATG	Bifidobacteria	Satokari et al. [35]
Reverse primers^d^			
Bif662-r	CCACCGTTACACCGGGAA	Bifidobacteria	Satokari et al. [35]
534r	5′ ATTACCGCGGCTGCTGG	Universal	Muyzer et al. [55]
Q-PCR primers^e^			
Bif spp for	TCGCGTCYGGTGTGAAAG	Bifidobacteria	Rinttilä et al. [56]
Bif spp rev	CCACATCCAGCRTCCAC	Bifidobacteria	Rinttilä et al. [56]
UniF	GTGSTGCAYGGYYGTCGTCA	Universal	Fuller et al. [57]
UniR	ACGTCRTCCMCNCCTTCCTC	Universal	Fuller et al. [57]
Bac303F	GAAGGTCCCCCACATTG	*Bacteroides* spp.	Bartosch et al. [58]
Bfr-Fmrev	CGCKACTTGGCTGGTTCAG	*Bacteroides* spp.	Ramirez-Farias et al. [51]
Erec482F	CGGTACCTGACTAAGAAGC	Cluster XIVa	Rinttilä et al. [56]
Erec870R	AGTTTYATTCTTGCGAACG	Cluster XIVa	Rinttilä et al. [56]

^a^Primer 27f was not used in this study but is shown for comparison and to indicate the positions of the three mismatches with the *Bifidobacteriales* 16S rRNA gene (in bold)

^b^Same as primer 7-f in Satokari et al. [35] and 27f-YM in Frank et al. [32]. Contains two degenerate positions but still has two mismatches with the *Bifidobacteriales* 16S rRNA gene (in bold). The fusion primer used also contained the 454 adaptor “A” sequence—see “Methods” section for full details

^c^27f-Chl—optimised for *Chlamydiales*; 27f-Bor—optimised for *Borrelia* group; 27f-Bif—optimised for *Bifidobacteriales*; 27f-Ato—optimised for *Atopobium* group. The fusion primers used also contained the 454 adaptor “A” sequence—see “Methods” section for full details

^d^The reverse primers for sequencing also contained the 454 adaptor “B” sequence and 12-base Golay barcodes. See “Methods” section and Additional file 1: Table S1 for full details

^e^The Q-PCR annealing temperatures used were 60 °C

**Fig. 1:**
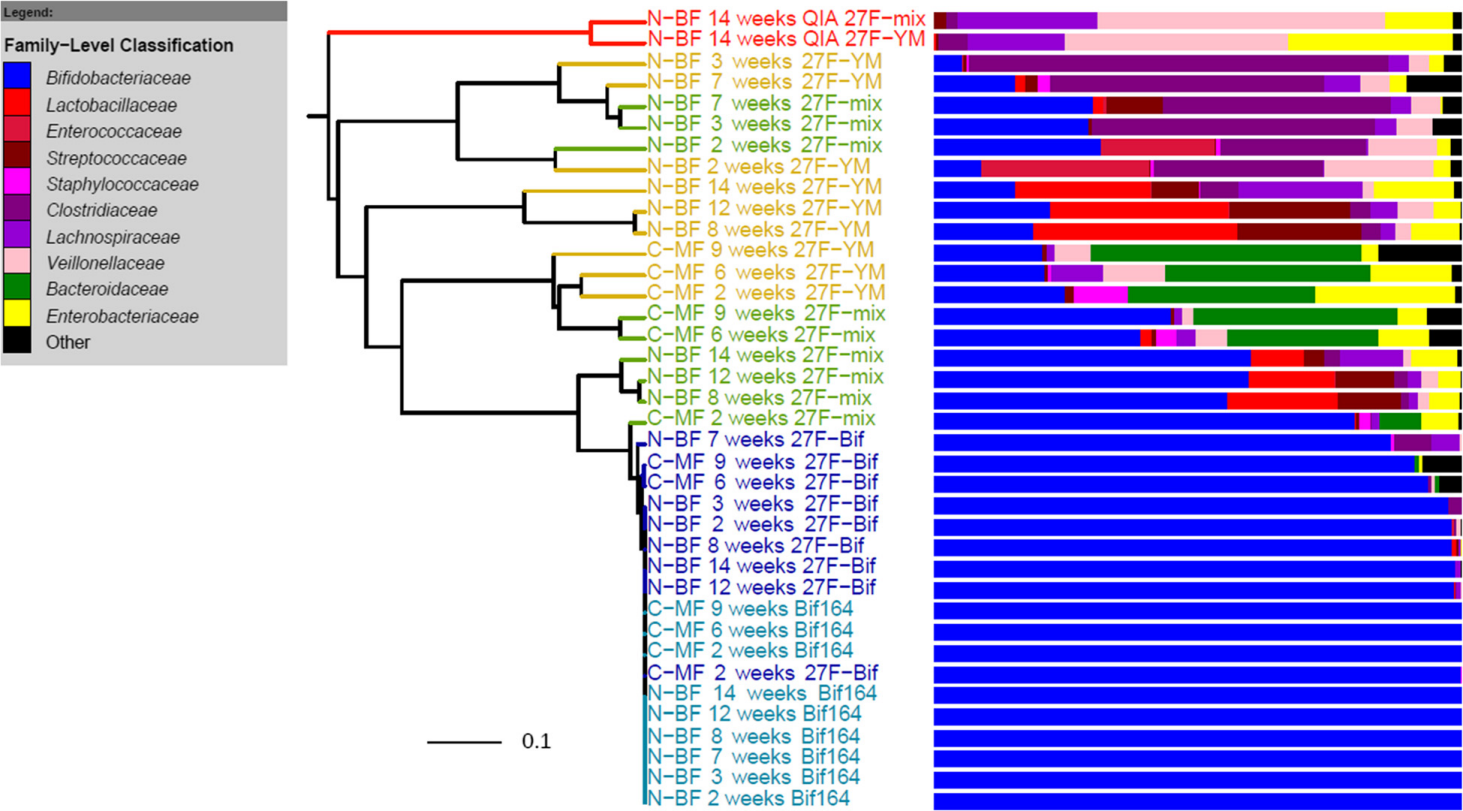
Dendrogram illustrating the microbial composition in two babies, pre-weaning. Thirty-eight sequenced samples are shown, derived from DNA extracted using the Fast DNA SPIN Kit for Soil, which contains a bead-beating step, from nine distinct samples from two babies at different time points, amplified with four primer sets (Table 2), and a further single DNA extraction of one sample using the, non-bead-beating, Qiagen QIAamp kit. *N*-*BF* indicates samples from the natural birth, solely breast-fed infant. *C*-*MF* indicates samples from the C-section birth, mixed-feeding infant. The infant age at time of sampling is shown (in weeks). The dendrogram clearly shows the difference in composition, specifically the lack of bifidobacterial sequences, between the Qiagen kit (marked with *QIA* and *red branches* in the figure) and every other sample. Different PCR primer combinations are indicated by branch colouring: *yellow*—27f-YM primer; *green*—27f-Mix combination of forward primers; the two shades of *blue* represent samples processed with the 27f-Bif and Bif164 control primer sets. *Adjacent bar charts* show the bacterial composition of the sequence data at the family level. Using the 27f-Mix PCR primers increased detection of bifidobacterial sequences compared to using the 27f-YM primer, which has two mismatches to the *Bifidobacterium* genus

In order to compare the effect of storage and DNA extraction method, DNA was extracted from a frozen aliquot of one faecal sample using both the FastDNA SPIN Kit for Soil (2-min bead-beating time; method 1) and the QIAamp DNA stool mini kit, which does not include a mechanical disruption step, following the manufacturer’s instructions (method 2). Using extraction method 1, the thawed faecal sample (stored frozen at −20 °C for 3 months) gave the same proportion of bifidobacteria as the original non-frozen, freshly extracted sample (Additional file 1: Figure S3). However, despite being the dominant genus when processed using method 1, no bifidobacteria were detected in the aliquot of this frozen sample when DNA was extracted using method 2 (the QIAamp DNA stool mini kit), regardless of which “universal” PCR primer sets were subsequently employed (Fig. 1). Indeed, the dominant bacterial family in the DNA sample extracted using method 2 was *Veillonellaceae*, comprising 40–50 % of the total bacteria detected, despite being a minor component (<5 %) in samples extracted using method 1 (Additional file 1: Figure S3). *Lactobacillaceae* were also undetectable when method 2 was used for DNA extraction despite otherwise comprising >25 % of the bacterial composition when DNA was extracted using method 1 prior to 16S rRNA gene sequence analysis. The relative increase in abundance of *Lactobacillaceae* in the sample that had been stored frozen was countered by a decrease in *Lachnospiraceae*. The choice of DNA extraction method therefore had a much greater effect on the apparent microbiota composition than did storage of the sample for 3 months at −20 °C prior to DNA extraction. Effective recovery of bifidobacterial sequences depends on the DNA extraction process incorporating a bead-beating step. The QIAamp kit, employed following the manufacturer’s extraction protocol, was clearly inadequate for extracting DNA from faecal samples for the purpose of profiling the total bacterial community using 16S rRNA gene sequencing.

Analysis of 16S rRNA genes by sequencing relies on a PCR amplification step. Standard “universal” primers such as 27f are routinely used, often with the assumption that the amplification efficiency will be approximately the same for all bacteria. We tested this assumption by comparing the results generated using a standard primer set, an optimised mixed primer set, and bifidobacteria-specific primers (Table 1). The mixed forward primer set (27f-Mix; Table 2) contains five different forward primers, four of which are specific for different bacterial groups, including bifidobacteria, that have sequence mismatches with the generic forward primer 27f (Table 1) [32] and also with the improved primer 27f-YM [35], used here. The efficiency of the bifidobacteria-specific primer in this mix was verified by comparing the data generated using only this forward primer (27f-Bif) with that obtained using an established set of primers for bifidobacteria detection (Bif164f plus Bif662r; [35]).

**Table 2:** Mean percentage of bacteria in specific families detected following 454 sequence analysis using the different primer sets across all samples

	Primer sets			
Forward primer	27f-YM^a^	27f-Mix^b^	27f-Bif^c^	Bif164^d^
Reverse primer	534r	534r	534r	Bif662-r
Family	Percentage of bacteria detected per family			
*Bifidobacteriaceae*	17.19 ± 2.37	47.30 ± 4.26	95.53 ± 1.25	100 ± 0.000001
*Lactobacillaceae*	16.03 ± 4.61	8.59 ± 2.58	0.07 ± 0.05	0 ± 0.00
*Enterococcaceae*	2.01 ± 1.71	1.31 ± 1.13	0.02 ± 0.02	0 ± 0.00
*Streptococcaceae*	9.41 ± 2.43	4.83 ± 1.08	0.11 ± 0.07	0 ± 0.00
*Staphylococcaceae*	1.07 ± 0.56	0.44 ± 0.22	0.04 ± 0.04	0 ± 0.00
*Clostridiaceae*	16.02 ± 5.27	10.65 ± 4.04	0.74 ± 0.48	0 ± 0.00
*Lachnospiraceae*	9.54 ± 2.40	8.60 ± 3.06	1.02 ± 0.43	0 ± 0.00
*Veillonellaceae*	5.38 ± 3.04	3.92 ± 0.91	0.13 ± 0.07	0 ± 0.00
*Bacteroidaceae*	10.15 ± 4.62	8.48 ± 3.74	0.24 ± 0.15	0 ± 0.00
*Enterobacteriaceae*	10.62 ± 1.90	3.80 ± 0.66	0.04 ± 0.03	0 ± 0.00
Other	2.56 ± 0.95	2.07 ± 0.56	2.05 ± 0.97	0 ± 0.00

Values given are the mean of all data, plus or minus SE of the mean. Data results from analysis of samples from two babies ages 2–14 weeks, at 17 time points, extracted using method 1 (FastDNA SPIN Kit for Soil) and amplified with four primer sets giving 68 sequence datasets. Average number of sequences per sample = 1645

^a^The degenerate primer 27f-YM sequence has two mismatches with the *Bifidobacterium* genus

^b^27f-Mix—4:1:1:1:1 molar mix of forward primers (27f-YM, 27f-Chl, 27f-Bor, 27f-Bif, 27f-Ato, respectively)

^c^Specific for bifidobacteria but designed to have similar amplification efficiency as the other primers

^d^Standard specific primers for detection of bifidobacteria

**Fig. 2:**
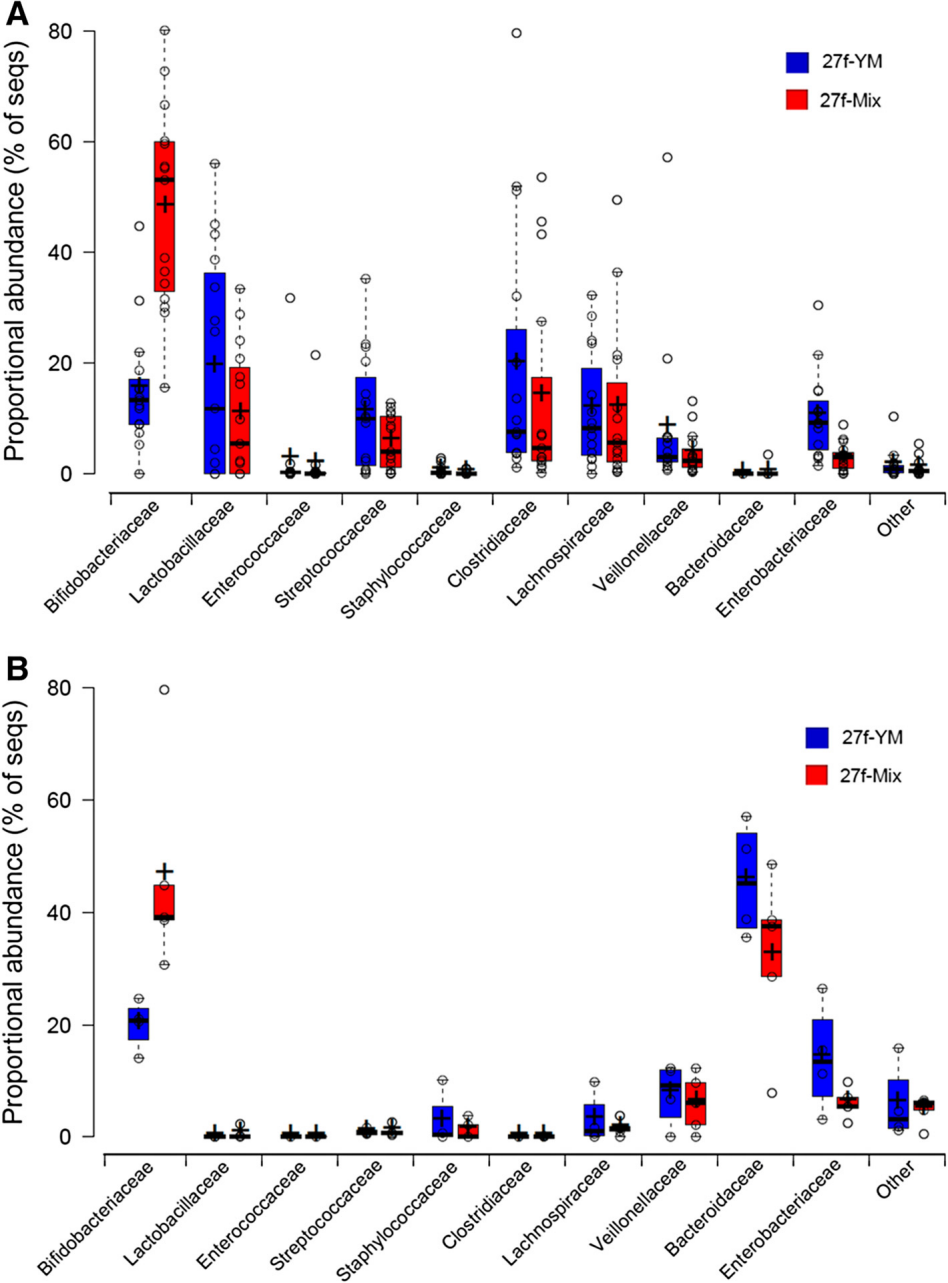
Comparison of bacterial families detected in faecal samples from two babies. Sequence data is based on 16S rRNA gene amplicons obtained using the 27f-YM (*blue*) or 27f-Mix (*red*) forward primers. **a** Baby N-BF: Data shows the mean percentage of sequences in each bacterial family after 15 separate DNA extractions at seven time points. **b** Baby C-MF: Data shows the mean percentage of sequences in each bacterial family after six separate DNA extractions at three time points. For both panels, individual data points are plotted as *open circles*; *centre lines* in the box plots show the medians; *crosses* represent sample means; *box limits* indicate the 25th and 75th percentiles as determined by R software; whiskers extend 1.5 times the interquartile range from the 25th and 75th percentiles, outliers are represented by *dots*. Plotted using BoxPlotR [52]

The compositional differences observed using the different primer sets were marked (Table 2, Fig. 1). As expected, the pyrosequencing 16S rRNA gene data generated using the primer combinations specific for bifidobacteria (27f-Bif/534r or Bif164f/Bif662r; Table 1) resulted in most of the sequences obtained being derived from *Bifidobacterium* species. All sequences amplified using the established bifidobacteria-specific primer set (Bif164/Bif662r) corresponded to bifidobacteria, while the 27f-Bif/534r combination was slightly less specific, with 4.5 % of the resulting sequences not identified as bifidobacteria (Table 2). The mixed forward primer set (27f-Mix) picked up on average 30 % more bifidobacteria than the single 27f-YM primer (Table 2), with a proportional reduction in some of the other bacterial genera enumerated. The specific profile generated from different babies clearly shows that the calculated proportional abundance of bifidobacteria depends not only on inter-individual variation but also crucially on primer choice. The use of the mixed forward primer (27f-Mix) significantly increased (at least doubling) the proportion of bifidobacteria detected compared to the single “universal” primer 27f-YM, while there was little difference in the detection of other bacterial genera (Fig. 2). Comparing the average proportional abundance of bifidobacteria in both babies at all ten time points, detected using the two primer sets, revealed that significantly fewer bifidobacteria sequences were detected with the basic primer 27f-YM (*p* < 0.001).

### Longitudinal bacterial diversity in two babies—comparing 16S rRNA gene pyrosequencing and fluorescent in situ hybridisation (FISH) data

Selected faecal samples from two of the babies, pre-weaning, were analysed by both 16S rRNA gene pyrosequencing and FISH in order to compare the bacterial composition detected using the two techniques. Reassuringly, the data generated using both techniques showed considerable overlap in the taxa that were detected, despite the fact that both techniques have distinct inherent advantages and biases. 16S rRNA gene sequencing can only be considered semi-quantitative due to factors such as rRNA operon copy number variation, and as such, data must be presented as proportional abundances rather than absolute abundances. FISH data has the advantage of enabling the actual bacterial load in the samples to be estimated. Even the very early 2-week samples contained more than 10^9^ bacteria/g faeces, and although there were some fluctuations, numbers remained relatively constant throughout the pre-weaning period (Additional file 1: Figure S4).

**Fig. 3:**
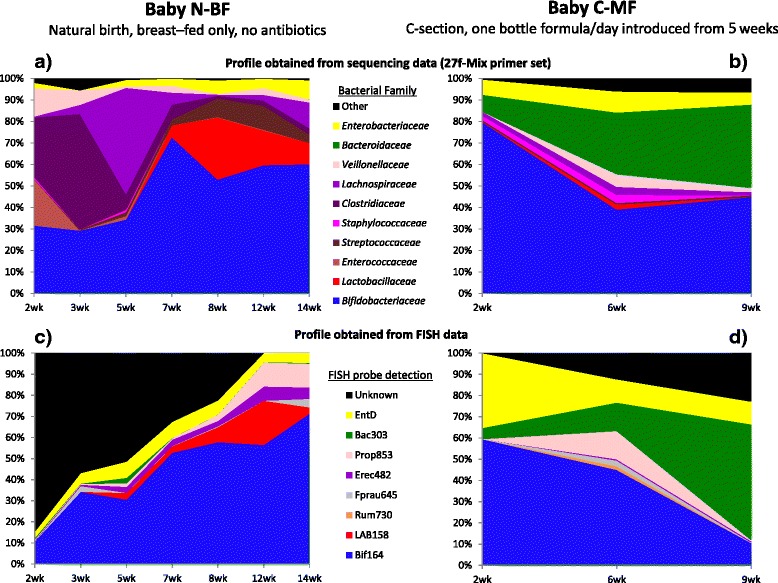
Longitudinal bacterial profile of two babies (pre-weaning), comparing FISH and 16S rRNA gene sequencing data. **a**, **b**—sequencing data (27f-Mix primer set); **c**, **d**—FISH data. **a**, **c** Baby N-BF, natural birth, breast-fed only; **b**, **d** Baby C-MF, C-section, one bottle formula/day introduced from 5 weeks. FISH probes used were Eub338 (total bacterial count), Erec482 (*Lachnospiraceae*), Fprau645 (*F. prausnitzii* group of the *Ruminococcaceae*), Bif164 (*Bifidobacterium* genus), Rum730 (Rfla729 + Rbro730) (*Ruminococcus flavefaciens* and *R. bromii* subclusters of the *Ruminococcaceae*), Prop853 (*Veillonellaceae*), Bac303 (*Bacteroides*-*Prevotella* group), LAB158 (*Lactobacillaceae* and *Enterococcaceae*) and EntD (*Enterobacteriaceae*). The same colouring scheme has been used to illustrate overlap between bacterial taxa identified using the two methods

The two babies had very different bacterial profiles, and it took between 3 and 7 weeks for the infant microbiota to stabilise. Although the panel of FISH probes had previously been shown to cover 80 % of the microbial species present in adult faecal samples [36], more than 50 % of the bacteria were unidentified in early samples from baby N-BF (Fig. 3c; Additional file 1: Figure S5). The population of bifidobacteria increased steadily to the 14-week time point, when approximately 60 % of the bacteria present in baby N-BF were bifidobacteria and *Bacteroides* populations remained undetectable (Fig. 3a, c). In contrast, with baby C-MF, the maximum population of bifidobacteria (>60 %) was detected at the 2- and 4-week time points (Fig. 3b, d; Additional file 1: Figure S5). During the transitional 5-week period between the introduction of formula-feeding and the complete withdrawal of breast milk, the bifidobacteria population decreased finally representing less than 10 % of the total microbiota (Additional file 1: Figure S5), while *Bacteroides* species became prevalent by 9 weeks and were maintained at >50 % of the total population until just before weaning. These findings are broadly consistent with previous studies of formula-fed versus breast-fed infants [7, 9, 37, 38].

The 16S rRNA gene sequence data using the 27f-Mix/534r primer combination revealed the “missing” bacterial diversity in the samples that was not detected using FISH. It was particularly useful in determining the bacterial species present in the 2- to 5-week samples from baby N-BF, when less than 50 % of the bacteria present had been detected using the standard set of FISH probes (Fig. 3c). The sequencing data indicated that these early samples were characterised by spikes in specific bacterial groups. For instance, the 2-week sample still contained >20 % of *Enterococcaceae* (99 % similarity to *Enterococcus faecalis*) and 15 % *Veillonellaceae* (100 % similarity to *Veillonella* spp.). These bacteria are among the early colonisers that create the anoxic conditions in the gut, prior to colonisation with more obligately anaerobic bacteria [7, 9]. The family *Clostridiaceae* formed between 20 and 30 % of the total microbial composition in the 2- to 5-week samples. However, sequence data classification showed an early abundance (20 %) of bacteria related to *Clostridium perfringens* (98 % similarity) being replaced by >40 % *Clostridium butyricum*-like species (98 % similarity) in the 5-week sample (Additional file 1: Table S2). Neither of these *Clostridium* species would have been detected with the specific FISH probes used. Although these species can be associated with an “unhealthy” gut status in adults, in contrast, there are a number of reports indicating the common presence of such bacteria, and even of C*lostridium difficile*, in seemingly healthy infants [12, 39, 40].

The bifidobacteria detected using the bifidobacteria-specific primer sets split between two operational taxonomic units (OTUs)—99.7 % *Bifidobacterium longum* and 0.3 % *Bifidobacterium adolescentis* (Additional file 1: Table S2). This correlates with the known abundance of the *B. longum* group, which includes *B. longum* subsp. *infantis*, in the infant microbiota [24]. In the purely breast-fed baby, >99 % of the total bifidobacteria sequences had a 100 % sequence match to *B. longum*. However, in the mixed-fed baby (C-MF), this was not the only *Bifidobacterium* species represented. *B. adolescentis*, commonly associated with adults but also identified in infants [41], formed >10 % of the bifidobacterial sequences from week 6 onwards, coinciding with the introduction of formula-feeding.

## Conclusions

Here, we present further evidence that the under-representation of *Actinobacteria* in many culture-independent analyses of the gut microbiota is a consequence of poor DNA extraction techniques, poor PCR primer choice or a combination of both. This issue applies equally to adult samples, where bifidobacteria and other *Actinobacteria* typically comprise less than 10 % of the microbiota [18–21, 42], but it becomes critically important when studying infants, particularly breast-fed infants, where the *Bifidobacterium* genus normally comprises in excess of 50 % of the microbiota. For example, a previous study indicated that bifidobacteria were a minor component of the faecal microbiota in both infants and adults [23], a finding that was in stark contrast to numerous other studies. Here, we clearly demonstrate that DNA extracted using method 2 (the QIAamp DNA extraction kit used by Palmer et al. [23]) contains no detectable bifidobacterial DNA sequences, whereas the same sample, extracted using a different method involving mechanical lysis, was dominated by bifidobacteria.

Several studies have now been published that provide evidence consistent with the present findings [43]. Maukonen et al. performed a detailed study comparing various commercial DNA extraction kits (including the two used here), concluding that numbers of bifidobacteria were as much as three logs higher when the DNA extraction method included a mechanical cell lysis step rather than simply an enzymatic process [25]. Interestingly, they also showed that the composition within both the *Lachnospiraceae* and *Ruminococcaceae* families was influenced by whether enzymatic or mechanical lysis preceded DNA extraction [25]. Different microbiota profiles for the same sample have also been reported between different kits that rely on mechanical disruption [27]. Ultimately, the optimal DNA extraction method has to be a balance between extracting as much DNA as possible from the sample without biasing the extraction towards particular taxa. Of five DNA extraction kits compared by Claassen et al. [26], each was “best” at facilitating detection of a different bacterial genus of the three genera they focussed on.

The chosen PCR primer sequences are also critical determinants of the final bacterial sequence profiles. It was previously demonstrated that degenerate primers are necessary for effective recovery of bifidobacterial sequences using variable regions V3–V5 of the 16S rRNA gene [33]. Here, we show that bacterial primers for the commonly targeted V1–V3 regions of the 16S rRNA gene also require modifications for effective recovery of bifidobacteria. The primers used in many analyses of the composition of the gut microbiota comprise of only the 27f forward primer, which, as shown here and in other publications, clearly has a bias towards poorer amplification of *Actinobacteria* (and thus *Bifidobacterium*) compared to other bacterial genera [28, 32]. Although it has been known for more than 20 years that universal primers targeting the 16S rRNA gene have limitations related to binding and amplification bias (reviewed by [34]), again some compromise may be necessary to detect as much of the bacterial diversity as possible. Combining the degenerate 27f-YM primer with additional primers, specifically modified to target groups with mismatches to the 27f sequence overcame the problem of under-representation of these bacteria. To avoid the inconvenience of ordering multiple forward primers and then mixing prior to PCR amplification, a primer configuration of AGMGTTYGATYMTGGCTCAG would widen specificity in the same way as the mixed primer set used here, compared to the commonly used 27f primer that has no degenerate bases.

The microbial composition we observed in the single breast-fed compared to formula-fed baby is in agreement with the majority of the literature on this subject. In previous studies, the abundance of *Bacteroides* was found to be greater relative to bifidobacteria in formula-fed infants [11], and a rapid increase was reported in the diversity of the microbiota following the introduction of a single bottle of formula-feeding [7]. In the data presented here, gaps in the bacterial composition apparent in the FISH data were identified using the mixed primer 16S rRNA gene sequencing data, while the FISH data confirmed the relative abundance of the bifidobacteria genus in the respective samples from the two babies. Thus, this work emphasises the critical impact sample processing methodology has on sequence data and shows how the use of multiple detection methods enables results to be cross-validated, giving additional confidence in the data generated.

## Methods

### Volunteer recruitment

Babies born to staff or friends of staff at the Rowett Institute were recruited and stool samples collected from nappies provided by the parents on a 1–2 weekly basis, prior to weaning. The detailed study of two babies presented here was part of a larger ongoing study, carried out with full ethical approval (study number 08/001—RINH Human Studies Ethical Review Committee). Full written consent was obtained from at least one parent prior to collection of any samples. Samples were stored at 4 °C and processed within 6 h of defaecation. Baby N-BF was a natural birth, and was exclusively breast-fed until weaning. Baby C-MF was a C-section birth and had mixed-feeding (one bottle of formula milk per day was introduced from 5 weeks old, with exclusive breast-feeding prior to that and exclusive formula-feeding from 11 weeks old). Neither baby received antibiotics during the study period.

### Sample processing

All faecal samples were initially placed inside a sterile plastic bag and hand-homogenised to a uniform consistency, and DNA was routinely extracted directly from 0.3 g of this fresh faecal material using the FastDNA SPIN Kit for Soil (MP Biomedicals), following the manufacturer’s instructions. In order to compare certain parameters of the extraction procedure, in some cases, DNA was extracted from both 0.3 and 0.5 g aliquots, either immediately or from frozen aliquots, and the sample was homogenised for either 30 s, 2 min or 5 min (in 30 s bursts, with intermittent cooling on ice). Assessing DNA yield using the Nanodrop (Nanodrop ND-1000 Spectrophotometer, Thermo Scientific) indicated that more than twice as much DNA (251 ng/μl compared to 108 ng/μl and 111 ng/μl compared to 46 ng/μl for the two samples tested) was obtained using the smaller, 0.3 g, starting samples, and this weight was subsequently routinely used. Finally, DNA was also extracted from a subset of identical samples using the widely used QIAamp DNA stool Mini Kit (QIAGEN no. 51504), directly following the manufacturer’s protocol.

Extracted DNA was used as a template for PCR amplification of bacterial 16S rRNA genes (four 25 μl reactions per sample, using 2 μl DNA per 25 μl reaction). Various different PCR primer combinations, also incorporating 12-mer Golay barcodes and 454 adaptor sequences to allow multiplexing and sequencing on the 454 sequencing platform using the Lib-L sequencing kit, were used (Tables 1 and 2). In brief, samples amplified with “27f-YM” used a single forward primer (*CCTATCCCCTGTGTGCCTTGGCAGTCTCAG*AGAGTTTGATYMTGGCTCAG, where the letters in italics show the 454 Lib-L “B” adaptor sequence and those in normal font show the 16S rRNA gene binding sequence), those with “27f-Mix” used a combination of five forward primers; 27f-YM (configuration as shown above), 27f-Chl (*CCTATCCCCTGTGTGCCTTGGCAGTCTCAG*AGAATTTGATCTTGGTTCAG), 27f-Bor (*CCTATCCCCTGTGTGCCTTGGCAGTCTCAG*AGAGTTTGATCCTGGCTTAG), 27f-Bif (*CCTATCCCCTGTGTGCCTTGGCAGTCTCAG*AGGGTTCGATTCTGGCTCAG), 27f-Ato (*CCTATCCCCTGTGTGCCTTGGCAGTCTCAG*AGAGTTCGATCCTGGCTCAG) and those with “27f-Bif” used a single forward primer over the same priming region as the other 27f primers that is optimised for the *Bifidobacteriales* group (configuration as shown for 27f-Bif above). All combinations of the 27f primer were used in conjunction with a fusion version of primer 534r (*CCATCTCATCCCTGCGTGTCTCCGACTCAG*-barcode-ATTACCGCGGCTGCTGG, where the letters in normal font show the 16S rRNA gene priming region, those in italics show 454 Lib-L adaptor “A”, and “-barcode-” indicates where individual unique 12-base Golay barcodes were used for each sample). As a further control, a bifidobacteria-specific primer set was also included. The forward primer was Bif164f (*CCTATCCCCTGTGTGCCTTGGCAGTCTCAG*GGGTGGTAATGCCGGATG, where the letters in italics show the 454 Lib-L “B” adaptor sequence and those in normal font show the bifidobacterial 16S rRNA gene binding sequence), and the reverse primer was Bif662r (*CCATCTCATCCCTGCGTGTCTCCGACTCAG*-barcode-CCACCGTTACACCGGGAA, where the letters in normal font show the bifidobacterial 16S rRNA gene priming region, those in italics show 454 Lib-L adaptor “A”, and “-barcode-” indicates where individual unique 12-base Golay barcodes were used for each sample). The Golay barcodes used for each of the sequenced samples are listed in Additional file 1: Table S1.

For Q-PCR amplification, extracted DNA was diluted to a concentration of 5 ng/μl in 5 ng/μl herring sperm DNA and amplified, in duplicate, using either universal bacterial primers or group-specific primers (Table 1). The amplification mix contained 2 μl DNA, 5 μl SYBR green ready mix (SIGMA 172–5121), 0.5 μl each primer (concentration 10 pmol/μl) and 2 μl sterile water. Amplification conditions were 1 cycle of 95 °C for 3 min, and 40 cycles of 95 °C for 5 s and 60 °C for 30 s using a Bio-Rad CFX 384 Real-time system. A final melt curve analysis was done with an incremental temperature increase of 0.5 °C every 5 s from 65 °C to 95 °C. Relative bacterial concentrations in each sample were estimated by comparing the gene copy numbers calculated using standard curves prepared with appropriate control DNA (starting concentration 16.4 pmol/μl).

### 16S rRNA gene sequence analysis

The sequences were analysed using the mothur software package [44]. In brief, the data was first filtered using the “trim.seqs” command, where the reads were truncated once average quality scores dropped below 35 across a rolling window of 50 bases. All reads that were less than 200 bp in length, that had any mismatches to either the primer or barcode sequences or that had ambiguous base calls or had homopolymeric stretches of longer than 8 bases were removed. We then used the “chimera.perseus” command in mothur to check for and then remove putative chimeric reads [45]. Following these quality control steps, a total of 110,642 sequences remained (median of 850 per sample, mean of 1558 per sample, range 14 to 6194). We calculated the Good’s coverage estimates for all of the samples using mothur. This revealed that the average (mean) coverage for all samples was 99.2 % (standard deviation of 1.9 %), and the median coverage was 99.8 %. We were therefore able to make accurate comparisons between the babies, despite the differential read depth.

The refined set of sequences was then aligned to the reference SILVA database provided in mothur, a distance matrix generated, and then, OTUs were generated by clustering sequences using the average neighbour setting in mothur at 97 % similarity. Each OTU was assigned a taxonomic classification at all levels from phylum to genus using the reference Ribosomal Database Project (RDP) database provided in mothur. A cluster dendrogram, using the Yue and Clayton calculator, was generated in mothur from the family-level classification data and was visualised using the iTOL web package [46]. *Bifidobacteriaceae* data generated using the 27f-YM and 27f-Mix primers were compared in detail by ANOVA with baby and week nested within baby as random effects and with primer as fixed effect.

### Bacterial enumeration using FISH

Fresh faecal samples (0.5 g aliquot) were fixed in paraformaldehyde [47] prior to using 16S rRNA-targeted fluorescent probes to detect the predominant groups of human faecal bacteria. The probes used were Eub338 (total bacterial count), Erec482 (*Lachnospiraceae*), Fprau645 (*Faecalibacterium prausnitzii* group), Bif164 (*Bifidobacterium* genus), Rfla729 + Rbro730 (*Ruminococcus flavefaciens* and *Ruminococcus bromii* subclusters), Prop853 (*Veillonellaceae*), Bac303 (*Bacteroides*-*Prevotella* group), LAB158 (*Lactobacillaceae* and *Enterococcaceae*) and EntD (*Enterobacteriaceae*). These probes have all been validated previously [36, 48, 49], and hybridisation was carried out using standard methods [47, 50, 53]. Cells were counted automatically using Cell^F software linked to an Olympus BX61 upright fluorescent microscope (Olympus UK Ltd).

### Availability of supporting data

Raw sequence data is available from the European Nucleotide Archive, under study accession numbers ERP005250 and ERP004372/sample accession numbers ERS421602 and ERS373498 (see Additional file 1: Table S1 for barcode information). Further supplementary data is available in additional files linked to this article.

## Additional file

Additional file 1:
**Supplemental figures and tables.** **Figure S1.** 16S rRNA gene copy number calculated for each Q-PCR primer set, applying different bead-beating times. **Figure S2.** Proportional abundances of different bacterial phyla (A) and genera (B) detected using the 27f-Mix primer set, on DNA extracted using the FastDNA SPIN Kit for Soil, applying different bead-beating times. **Figure S3.** Effect of DNA extraction method and sample storage on bacterial profile detected using primer sets 27f-YM and 27f-Mix. **Figure S4.** Total Bacterial count in pre-weaned babies, detected using FISH probe Eub338. **Figure S5.** Bacteria detected in pre-weaning samples from two babies by FISH. **Table S1.** Golay barcode tags used for 16S rRNA gene pyrosequencing. **Table S2.** Proportional abundance (in %) of each OTU per sample (97 % OTU cut-off).

## Abbreviations

DGGE: denaturing gradient gel electrophoresis
FISH: fluorescent in situ hybridisation
OTUs: operational taxonomic units
Q-PCR: quantitative real-time PCR
