# Supplementary material for: 16S rRNA gene-based profiling of the human infant gut microbiota is strongly influenced by sample processing and PCR primer choice
Source: Microbiome. 2015 Jun 22;3:26. doi: 10.1186/s40168-015-0087-4 (PMC4482049; doi:10.1186/s40168-015-0087-4)
Supplement: Additional file 1: — Supplemental figures and tables. Figure S1. 16S rRNA gene copy number calculated for each Q-PCR primer set, applying different bead-beating times. Figure S2. Proportional abundances of different bacterial phyla (A) and genera (B) detected using the 27f-Mix primer set, on DNA extracted using the FastDNA SPIN Kit for Soil, applying different bead-beating times. Figure S3. Effect of DNA extraction method and sample storage on bacterial profile detected using primer sets 27f-YM and 27f-Mix. Figure S4. Total Bacterial count in pre-weaned babies, detected using FISH probe Eub338. Figure S5. Bacteria detected in pre-weaning samples from two babies by FISH. Table S1. Golay barcode tags used for 16S rRNA gene pyrosequencing. Table S2. Proportional abundance (in %) of each OTU per sample (97 % OTU cut-off). [file 40168_2015_87_MOESM1_ESM.docx]

**16S rRNA gene-based profiling of the human infant gut microbiota is strongly influenced by sample processing and PCR primer choice**

**Supplementary Figures only**

**Supplementary Figure 1**

16S rRNA gene copy number calculated for each Q-PCR primer set using 5ng input DNA extracted from 0.3 g of the 5 week baby N-BF sample.

Bead beating was carried out for 30 sec bursts, for a total of 30 sec, 2 min or 5 min, as indicated.

Q-PCR primer sequences are given in Table 1.

**Supplementary Figure 2a**

Proportional abundances of different bacterial phyla detected using the 27f-Mix primer set, on DNA extracted using the FastDNA SPIN Kit for Soil, applying different bead-beating times.

Baby N-BF – natural birth, exclusive breast feeding, 7 week sample

Baby C-MF – caesarian section, mixed feeding, 9 week sample

**Supplementary Figure 2b**

Proportional abundances of different bacterial genera detected using the 27f-Mix primer set, on DNA extracted using the FastDNA SPIN Kit for Soil, applying different bead-beating times. Comparison of the proportions in i) baby N-BF and ii) baby C-MF after bead-beating times of 30 sec (red), 2 min (blue) and 5 min (green).

i) baby N-BF ii) baby C-MF

**Supplementary Figure 3**

Effect of DNA extraction method and sample storage on bacterial profile detected using primer sets 27f-YM and 27f-Mix

Comparison of the relative percentages of bacterial species present in the same sample (Baby N-BF, 14 week sample), extracted using either the FastDNA SPIN Kit for Soil (method 1) on two occasions - fresh or frozen (3 months) or once using the QIAamp DNA stool mini kit (method 2; frozen sample).

**Supplementary Figure 4** Total Bacterial count in pre-weaned babies, detected using FISH probe Eub338


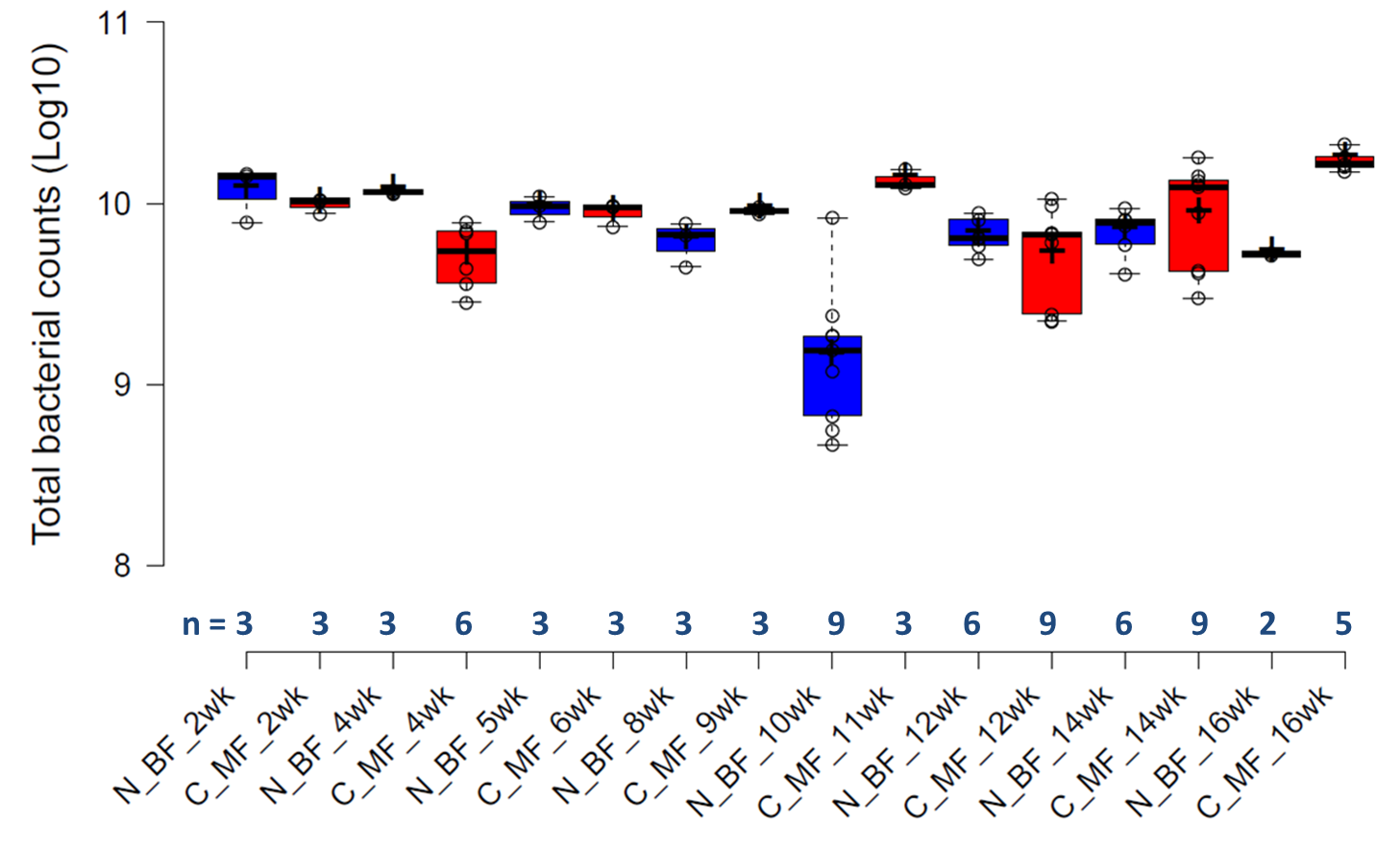


Range of log10 converted total bacterial FISH counts (using probe Eub338) in samples from baby N-BF (blue) and baby C-MF (red) at sampling points as indicated by age in weeks in the sample names. Centre lines show the medians; crosses represent sample means; box limits indicate the 25th and 75th percentiles as determined by R software; whiskers extend 1.5 times the interquartile range from the 25th and 75th percentiles, outliers are represented by dots; data points are plotted as open circles. Plotted using BoxPlotR.

Reference:

Spitzer,M.; Wildenhain, J.; Rappsilber, J.; Tyers, M. 2014. BoxPlotR: a web tool for generation of box plots. Nature Methods 11, 121–122.

**Supplementary Figure 5** Bacteria detected in pre-weaning samples from two babies by FISH. Data are the mean of at least three separate enumerations, and the standard error is shown.

**Baby N-BF –** breast fed throughout study period, no antibiotics

**Baby C-MF –** breast fed for the first four weeks, single bottle of formula introduced per day from 5 weeks old, full formula feeding from week 11. Baby received no antibiotics, but mother on trimethoprim treatment when baby 2 – 4 weeks old.

**Supplementary Table 1**– Golay barcode tags used for 16S rRNA gene pyrosequencing

| **ENA Study Accession Number** | **ENA Sample Accession Number** |  |  |  | **ENA Study Accession Number** | **ENA Sample Accession Number** |
| --- | --- | --- | --- | --- | --- | --- |
| **ERP005250** | **ERS421602** |  |  |  | **ERP004372** | **ERS373498** |
|  |  |  |  |  |  |  |
| **Sample** | **Barcode** | **Sample weight used** | **Bead-beating time** | **Primer Combination** | **Barcode** |  |
| **C-MF_2_weeks_27F-YM** | AACGCACGCTAG | 0.5 g | 30 sec | 27f-YM/534r |  |  |
| **C-MF_2_weeks_27F-mix** | ACACTGTTCATG | 0.5 g | 30 sec | 27f-mix/534r | ACACTGTTCATG | (Repeated sequencing to increase read depth) |
| **C-MF_2_weeks_27F-Bif** | ACCAGACGATGC | 0.5 g | 30 sec | 27f-Bif/534r |  |  |
| **C-MF_2_weeks_Bif164** | ACGCTCATGGAT | 0.5 g | 30 sec | Bif164/Bif662 |  |  |
| **C-MF_6_weeks_27F-YM** | ACTCACGGTATG | 0.5 g | 30 sec | 27f-YM/534r | ACTCACGGTATG | (Repeated sequencing to increase read depth) |
| **C-MF_6_weeks_27F-mix** | AGACCGTCAGAC | 0.5 g | 30 sec | 27f-mix/534r | AGACCGTCAGAC | (Repeated sequencing to increase read depth) |
| **C-MF_6_weeks_27F-Bif** | AGCACGAGCCTA | 0.5 g | 30 sec | 27f-Bif/534r | AGCACGAGCCTA | (Repeated sequencing to increase read depth) |
| **C-MF_6_weeks_Bif164** | ACAGACCACTCA | 0.5 g | 30 sec | Bif164/Bif662 | ACAGACCACTCA | (Repeated sequencing to increase read depth) |
| **C-MF_9_weeks_27F-YM** | ACCAGCGACTAG | 0.5 g | 30 sec | 27f-YM/534r |  |  |
| **C-MF_9_weeks_27F-mix** | ACGGATCGTCAG | 0.5 g | 30 sec | 27f-mix/534r |  |  |
| **C-MF_9_weeks_27F-Bif** | AGCTTGACAGCT | 0.5 g | 30 sec | 27f-Bif/534r |  |  |
| **C-MF_9_weeks_Bif164** | AACTGTGCGTAC | 0.5 g | 30 sec | Bif164/Bif662 |  |  |
| C-MF_9_weeks_27F | ACCGCAGAGTCA | 0.5 g | 2 min | 27f-YM/534r | ACCGCAGAGTCA | (Repeated sequencing to increase read depth) |
| C-MF_9_weeks_27F-mix | ACGGTGAGTGTC | 0.5 g | 2 min | 27f-mix/534r | ACGGTGAGTGTC | (Repeated sequencing to increase read depth) |
| C-MF_9_weeks_27F-Bif | ACTCGATTCGAT | 0.5 g | 2 min | 27f-Bif/534r | ACTCGATTCGAT | (Repeated sequencing to increase read depth) |
| C-MF_9_weeks_Bif164 | AGACTGCGTACT | 0.5 g | 2 min | Bif164/Bif662 | AGACTGCGTACT | (Repeated sequencing to increase read depth) |
| C-MF_9_weeks_27F-mix | AGGACGCACTGT | 0.5 g | 5 min | 27f-mix/534r |  |  |
| C-MF_9_weeks_27F-Bif | AAGAGATGTCGA | 0.5 g | 5 min | 27f-Bif/534r |  |  |
| C-MF_9_weeks_Bif164 | ACAGCAGTGGTC | 0.5 g | 5 min | Bif164/Bif662 | ACAGCAGTGGTC | (Repeated sequencing to increase read depth) |
| **N-BF_2_weeks_27F-YM** | ACGTACTCAGTG | 0.5 g | 30 sec | 27f-YM/534r |  |  |
| **N-BF_2_weeks_27F-mix** | ACTCGCACAGGA | 0.5 g | 30 sec | 27f-mix/534r |  |  |
| **N-BF_2_weeks_27F-Bif** | AGAGAGCAAGTG | 0.5 g | 30 sec | 27f-Bif/534r |  |  |
| **N-BF_2_weeks_Bif164** | AGCATATGAGAG | 0.5 g | 30 sec | Bif164/Bif662 |  |  |
| **N-BF_7_weeks_27F-YM** | AGGCTACACGAC | 0.5 g | 30 sec | 27f-YM/534r | AGGCTACACGAC | (Repeated sequencing to increase read depth) |
| **N-BF_7_weeks_27F-mix** | AAGCTGCAGTCG | 0.5 g | 30 sec | 27f-mix/534r | AAGCTGCAGTCG | (Repeated sequencing to increase read depth) |
| **N-BF_7_weeks_27F-Bif** | ACAGCTAGCTTG | 0.5 g | 30 sec | 27f-Bif/534r | ACAGCTAGCTTG | (Repeated sequencing to increase read depth) |
| **N-BF_7_weeks_Bif164** | ACCTGTCTCTCT | 0.5 g | 30 sec | Bif164/Bif662 | ACCTGTCTCTCT | (Repeated sequencing to increase read depth) |
| N-BF_7_weeks_27F | ACGTCTGTAGCA | 0.5 g | 2 min | 27f-YM/534r |  |  |
| N-BF_7_weeks_27-Fmix | AGAGCAAGAGCA | 0.5 g | 2 min | 27f-mix/534r |  |  |
| N-BF_7_weeks_27F-Bif | AGCCATACTGAC | 0.5 g | 2 min | 27f-Bif/534r |  |  |
| N-BF_7_weeks_Bif164 | AGGTGTGATCGC | 0.5 g | 2 min | Bif164/Bif662 |  |  |
| N-BF_7_weeks_27F | AATCAGTCTCGT | 0.5 g | 5 min | 27f-YM/534r |  |  |
| N-BF_7_weeks_27F-mix | ACGACGTCTTAG | 0.5 g | 5 min | 27f-mix/534r |  |  |
| N-BF_7_weeks_27F-Bif | ACGTGAGAGAAT | 0.5 g | 5 min | 27f-Bif/534r |  |  |
| N-BF_7_weeks_Bif164 | ACTGACAGCCAT | 0.5 g | 5 min | Bif164/Bif662 |  |  |
| **N-BF_3_weeks_27F-Bif** | AGCGACTGTGCA | 0.3 g | 2 min | 27f-YM/534r |  |  |
| **N-BF_3_weeks_27F-YM** | AGTACGCTCGAG | 0.3 g | 2 min | 27f-mix/534r |  |  |
| **N-BF_3_weeks_27F-mix** | AATCGTGACTCG | 0.3 g | 2 min | 27f-Bif/534r |  |  |
| **N-BF_3_weeks_Bif164** | ACGAGTGCTATC | 0.3 g | 2 min | Bif164/Bif662 |  |  |
| **N-BF_8_weeks_27F-YM** | ACTGATCCTAGT | 0.5 g | 2 min | 27f-YM/534r |  |  |
| **N-BF_8_weeks_27F-mix** | AGAGTCCTGAGC | 0.5 g | 2 min | 27f-mix/534r |  |  |
| **N-BF_8_weeks_27F-Bif** | AGCGAGCTATCT | 0.5 g | 2 min | 27f-Bif/534r |  |  |
| **N-BF_8_weeks_Bif164** | AGTACTGCAGGC | 0.5 g | 2 min | Bif164/Bif662 |  |  |
| N-BF_8_weeks_fr_27F-Bif | ACACACTATGGC | frozen o/n then 0.3 g | 5 min | 27f-YM/534r |  |  |
| N-BF_8_weeks_fr_27F | ACGATGCGACCA | frozen o/n then 0.3 g | 5 min | 27f-mix/534r |  |  |
| N-BF_8_weeks_fr_27F-mix | ACGTTAGCACAC | frozen o/n then 0.3 g | 5 min | 27f-Bif/534r |  |  |
| N-BF_8_weeks_fr_Bif164 | ACTGTACGCGTA | frozen o/n then 0.3 g | 5 min | Bif164/Bif662 |  |  |
| N-BF_8_weeks_27F | AGATACACGCGC | 0.3 g | 2 min | 27f-YM/534r |  |  |
| N-BF_8_weeks_27F-mix | AGCGCTGATGTG | 0.3 g | 2 min | 27f-mix/534r |  |  |
| N-BF_8_weeks_27F-Bif | ACACATGTCTAC | 0.3 g | 2 min | 27f-Bif/534r |  |  |
| N-BF_8_weeks_Bif164 | ACATGATCGTTC | 0.3 g | 2 min | Bif164/Bif662 |  |  |
| **N-BF_12_weeks_27F-YM** | ACGCAACTGCTA | 0.3 g | 2 min | 27f-YM/534r |  |  |
| **N-BF_12_weeks_27F-mix** | ACTGTCGAAGCT | 0.3 g | 2 min | 27f-mix/534r |  |  |
| **N-BF_12_weeks_27F-Bif** | AGCGTAGGTCGT | 0.3 g | 2 min | 27f-Bif/534r |  |  |
| **N-BF_12_weeks_Bif164** | ACACGAGCCACA | 0.3 g | 2 min | Bif164/Bif662 |  |  |
| **N-BF_14_weeks_27F-YM** | ACATGTCACGTG | 0.3 g | 2 min | 27f-YM/534r |  |  |
| **N-BF_14_weeks_27F-mix** | ACGCGATACTGG | 0.3 g | 2 min | 27f-mix/534r |  |  |
| **N-BF_14_weeks_27F-Bif** | ACTACGTGTGGT | 0.3 g | 2 min | 27f-Bif/534r |  |  |
| **N-BF_14_weeks_Bif164** | ACTGTGACTTCA | 0.3 g | 2 min | Bif164/Bif662 |  |  |
|  |  |  |  |  |  |  |
| **ENA Study Accession Number** | **ENA Sample Accession Number** |  |  |  |  |  |
| **ERP004372** | **ERS373498** |  |  |  |  |  |
|  |  |  |  |  |  |  |
| N-BF_14_weeks_27F | AGATACACGCGC | 0.3 g | 2 min | 27f-YM/534r |  |  |
| N-BF_14_weeks_27F-mix | AGCGCTGATGTG | 0.3 g | 2 min | 27f-mix/534r |  |  |
| N-BF_14_weeks_fr_27F | ACGCAACTGCTA | frozen 3 months then 0.3 g | 2 min | 27f-YM/534r |  |  |
| N-BF_14_weeks_fr_27F-mix | ACTGTCGAAGCT | frozen 3 months then 0.3 g | 2 min | 27f-mix/534r |  |  |
| **N-BF_14_weeks_QIAamp_27F-YM** | ACATGTCACGTG | frozen 3 months then 0.3 g | QIAamp | 27f-YM/534r |  |  |
| **N-BF_14_weeks_QIAamp_27F-mix** | ACGCGATACTGG | frozen 3 months then 0.3 g | QIAamp | 27f-mix/534r |  |  |
| N-BF_4 weeks_27F | AGCGACTGTGCA | 0.5 g | 30 sec | 27f-YM/534r |  |  |
| N-BF_4 weeks_27F-mix | AGTACGCTCGAG | 0.5 g | 30 sec | 27f-mix/534r |  |  |
| N-BF_5week_27F | ACTGATCCTAGT | 0.3 g | 30 sec | 27f-YM/534r |  |  |
| N-BF_5week_27F-mix | AGAGTCCTGAGC | 0.3 g | 30 sec | 27f-mix/534r |  |  |
| N-BF_5week_27F | ACACACTATGGC | 0.3 g | 2 min | 27f-YM/534r |  |  |
| N-BF_5week_27F-mix | ACGATGCGACCA | 0.3 g | 2 min | 27f-mix/534r |  |  |
| Blank control | ATACGTCTTCGA | No sample added | 30 sec | 27f-YM/534r |  |  |

**Footnote:**

Data were generated across two separate 454 pyrosequencing runs. The ENA accession numbers for each of these runs are indicated above all of the sample/barcode combinations that were included on each of them. Some samples were sequenced twice, in order to generate additional sequencing depth. Data from these replicates were pooled into single sequence libraries per sample. The barcodes that were used for these samples on the two sequencing runs are shown in the table. Samples highlighted in bold are those included in Figure 1.

**Supplementary Table 2** Proportional abundance (in %) of each OTU per sample (97% OTU cut-off)

|  | **Baby N-BF** (age in weeks) | | | | | | | **Baby C-MF** (age in weeks) | | |  |
| --- | --- | --- | --- | --- | --- | --- | --- | --- | --- | --- | --- |
| NCBI BLAST ID of closest cultured isolate | 2 wk | 3 wk | 5 wk | 7 wk | 8 wk | 12 wk | 14 wk | 2 wk | 6 wk | 9 wk | Total no. sequences |
| *Bifido longum* | 31.6 | 29.3 | 34.3 | 72.8 | 52.8 | 59.7 | 59.9 | 79.5 | 30.1 | 43.8 | 71554 |
| *Lactobacillus casei/paracasei* | 0.0 | 0.0 | 0.0 | 5.3 | 27.0 | 16.1 | 9.3 | 0.2 | 2.3 | 0.0 | 9746 |
| *Clost hathewayi* | 0.0 | 0.0 | 47.0 | 4.8 | 0.1 | 0.7 | 2.0 | 1.4 | 0.8 | 0.0 | 5420 |
| *Strep gallo/infant/mace* | 0.0 | 0.0 | 0.0 | 2.4 | 7.7 | 10.6 | 3.4 | 0.1 | 0.8 | 0.0 | 4319 |
| *Clost paraput/butyr* | 9.0 | 31.7 | 6.5 | 3.1 | 0.9 | 1.5 | 0.6 | 0.1 | 0.0 | 0.0 | 3909 |
| *Klebsiella pneumoniae* | 0.0 | 0.0 | 2.5 | 3.1 | 4.9 | 2.6 | 7.6 | 6.6 | 9.8 | 5.5 | 3135 |
| *Rum gauv/gnavus* | 0.4 | 4.3 | 2.4 | 0.9 | 0.2 | 1.9 | 9.8 | 0.1 | 0.0 | 0.0 | 2565 |
| *Veillonella parvula/dispar* | 13.2 | 6.7 | 0.7 | 3.1 | 0.2 | 3.2 | 1.5 | 0.0 | 6.0 | 2.2 | 2186 |
| *Clostridium perfringens* | 18.6 | 22.0 | 0.8 | 3.8 | 0.0 | 0.9 | 2.3 | 0.0 | 0.0 | 0.0 | 1961 |
| *Enterococcus faecalis* | 21.6 | 0.0 | 1.7 | 0.0 | 0.1 | 0.3 | 0.0 | 0.0 | 0.0 | 0.0 | 1433 |
| *Bact ster/clarus/unif* | 0.0 | 0.0 | 0.0 | 0.0 | 0.0 | 0.0 | 0.0 | 5.5 | 20.3 | 32.8 | 920 |
| *Citrobact amalon/farmer* | 0.0 | 0.0 | 0.2 | 0.2 | 0.5 | 1.5 | 0.9 | 0.0 | 0.0 | 0.0 | 434 |
| *Staphylococcus epidermidis* | 0.9 | 0.0 | 1.1 | 0.0 | 0.0 | 0.0 | 0.0 | 2.1 | 3.8 | 0.0 | 361 |
| *Serratia marcescens* | 2.5 | 0.0 | 0.3 | 0.0 | 0.1 | 0.0 | 0.0 | 0.0 | 0.0 | 0.0 | 291 |
| *Strep thermo/saliv/vestib* | 0.1 | 0.6 | 0.5 | 0.0 | 0.3 | 0.2 | 0.1 | 0.6 | 0.0 | 0.0 | 252 |
| *Bacteroides vulgatus* | 0.0 | 0.0 | 0.0 | 0.0 | 0.0 | 0.0 | 0.0 | 2.2 | 4.5 | 2.6 | 232 |
| *Strep pneu/tigur/mitis/crist* | 0.1 | 0.0 | 1.0 | 0.0 | 0.0 | 0.2 | 0.2 | 0.0 | 0.0 | 0.7 | 229 |
| *Haemophilus parainfluenzae* | 1.8 | 4.9 | 0.0 | 0.0 | 0.0 | 0.0 | 0.0 | 0.0 | 0.0 | 0.0 | 215 |
| *Bifidobacterium adolescentis* | 0.0 | 0.0 | 0.0 | 0.0 | 0.0 | 0.0 | 0.0 | 0.0 | 9.0 | 1.1 | 199 |
| *Collinsella aerofaciens* | 0.0 | 0.0 | 0.0 | 0.0 | 0.0 | 0.0 | 0.0 | 0.0 | 6.0 | 5.8 | 176 |
| *Bacteroides caccae* | 0.0 | 0.0 | 0.0 | 0.0 | 0.0 | 0.0 | 0.0 | 0.1 | 3.8 | 3.3 | 75 |

*Bifido longum =* Bifidobacterium longum; *Clost hathewayi = Clostridium hathewayi; Strep gallo/infant/mace = Streptococcus gallolyticus/infantarius/macedonicus;*

*Clost paraput/butyr = Clostridium paraputrificum/butyricum*

*Rum gauv/gnavus = Ruminococcus gauvreauii/gnavus; Bact ster/clarus/unif = Bacteroides stercoris/clarus/uniformis*

*Citrobact amalon/farmer = Citrobacter amalonaticus/farmer; Strep thermo/saliv/vestib = Streptococcus thermophilus/salivarius/vestibularis*

*Strep pneu/tigur/mitis/crist = Streptococcus pneumoniae/tigurinus/mitis/cristatus*

Data generated using the 27f-Mix/534R primer combination. An average of 2125 (baby N-BF) and 419 (baby C-MF) sequences were obtained per sample
